# A Comprehensive Analysis of Fibromyalgia and the Role of the Endogenous Opioid System

**DOI:** 10.3390/biomedicines13010165

**Published:** 2025-01-11

**Authors:** Mario García-Domínguez

**Affiliations:** 1 Program of Immunology and Immunotherapy, CIMA-Universidad de Navarra, 31008 Pamplona, Spain; mgdom@unav.es; 2 Department of Immunology and Immunotherapy, Clínica Universidad de Navarra, 31008 Pamplona, Spain; 3 Centro de Investigación Biomédica en Red de Cáncer (CIBERONC), 28029 Madrid, Spain

**Keywords:** central nervous system sensitization, chronic fatigue, chronic pain, fibromyalgia, musculoskeletal pain, pain measurement, opioid peptides and receptors

## Abstract

Fibromyalgia represents a chronic pain disorder characterized by musculoskeletal pain, fatigue, and cognitive impairments. The exact mechanisms underlying fibromyalgia remain undefined; as a result, diagnosis and treatment present considerable challenges. On the other hand, the endogenous opioid system is believed to regulate pain intensity and emotional responses; hence, it might be expected to play a key role in the enhanced sensitivity experienced by fibromyalgia patients. One explanation for the emergence of disrupted pain modulation in individuals with fibromyalgia is a significant reduction in opioid receptor activity or an imbalance in the levels of endogenous opioid peptides. Further research is essential to clarify the complex details of the mechanisms underlying this abnormality. This complexity arises from the notion that an improved understanding could contribute to the development of innovative therapeutic strategies aimed at targeting the endogenous opioid system in the context of fibromyalgia. Although progress is being made, a complete understanding of these complexities remains a significant challenge. This paradigm has the potential to revolutionize the complex management of fibromyalgia, although its implementation may experience challenges. The effectiveness of this approach depends on multiple factors, but the implications could be profound. Despite the challenges involved in this transformation, the potential for improving patient care is considerable, as this condition has long been inadequately treated.

## 1. Introduction

Fibromyalgia is a chronic pain disorder characterized by widespread musculoskeletal pain accompanied by symptoms that severely limit the quality of life of those affected. Classic symptoms include chronic fatigue, which can range from mild lethargy to severe exhaustion, sleep disturbances (such as insomnia or non-restorative sleep), and cognitive deficits, commonly known as “fibro fog”, which affect memory and concentration (Table 1) [1]. Fibromyalgia is usually characterized by severe pain associated with increased tenderness in certain areas, designated as “tender points” by the American College of Rheumatology (ACR), which are sensitive to pressure. Pain is commonly persistent and widespread and is often exacerbated by factors such as stress, physical activity, and weather changes [2]. Other symptoms include fatigue [3], sleep disturbances [4], cognitive issues [5], mood disorders [6], sensory impairments [7], gastrointestinal disorders [8], morning stiffness [9], dizziness [10], restless legs syndrome (RLS) [11], and painful menstrual periods (known as dysmenorrhea) [12]. Some studies estimate the prevalence of fibromyalgia to be between 2% and 8%, impacting approximately 5 to 10 million adults in the United States [13], although the prevalence ranges from 2.4% to 3.3% in Europe and South America [14,15]. This fact indicates the importance of fibromyalgia as a public health problem.

The incidence of this pathology is higher in women compared to men, suggesting a role of gender-specific factors for its onset and development [16]. Even though the exact causes of fibromyalgia are not fully understood, novel studies suggest that the disease results from altered sensory processing combined with several factors (e.g., genetics and environment) [17,18]. Associations between abnormalities in the serotonin, noradrenaline, and dopamine pathways and the onset of fibromyalgia have been revealed in several genetic studies [17,19]. Other triggering factors include traumatic injuries, infections, and extreme psychological stress [20]. Many neurobiological studies have indicated that most individuals with fibromyalgia reveal central sensitization. This pathological mechanism shows an amplification of nociceptive input to the central nervous system (CNS), where responses occur in response to stimuli below the threshold level [21].

Recent investigations have identified the opioid system as a major factor in the development of fibromyalgia [22]. This physiological system consists of endogenous opioid peptides and their corresponding receptors distributed throughout the CNS, peripheral nervous system (PNS), and other peripheral tissues. Three subclasses of opioid receptors (mu -µ-, delta -δ-, and kappa -κ-) have been identified, which differ in their functions and distribution patterns [23]. Their endogenous ligands (β-endorphin -β-END-, enkephalins -ENKs-, and dynorphins -DYNs-) modulate numerous physiological functions ranging from pain relief to the improvement of emotional well-being [24,25,26]. Several studies have shown that fibromyalgia is associated with a hypoopioidergic state due to the inability of those affected to modulate pain. Consequently, their bodies might not produce enough opioid peptides, a phenomenon which can result in a modified perception of pain and an increased sensitivity to painful stimuli [27].

This article provides an in-depth analysis of the opioid system in the context of fibromyalgia, particularly the essential role, its influence on the development of fibromyalgia, and the implications for therapeutic strategies focused on managing this condition. Understanding the interactions between the opioid system and fibromyalgia is expected to improve our understanding of the disease, enable the development of novel treatments and enhance patient outcomes.

## 2. Overview of the Endogenous Opioid System

The opioid system is a physiological system that includes opioid receptors, endogenous opioid peptides, and the enzymes responsible for the synthesis and degradation [25]. This system has an important modulatory function in the onset and development of pain and emotions, as well as some physiological functions [25]. Disorders in the endogenous opioid system are involved in pain management, mood disorders, and addiction. Understanding the complexity of the opioid system is crucial for the development of effective therapeutic strategies, especially for overcoming the issues associated with opioid abuse and physical dependence [28].

### 2.1. Opioid Receptors

**Figure 1:**
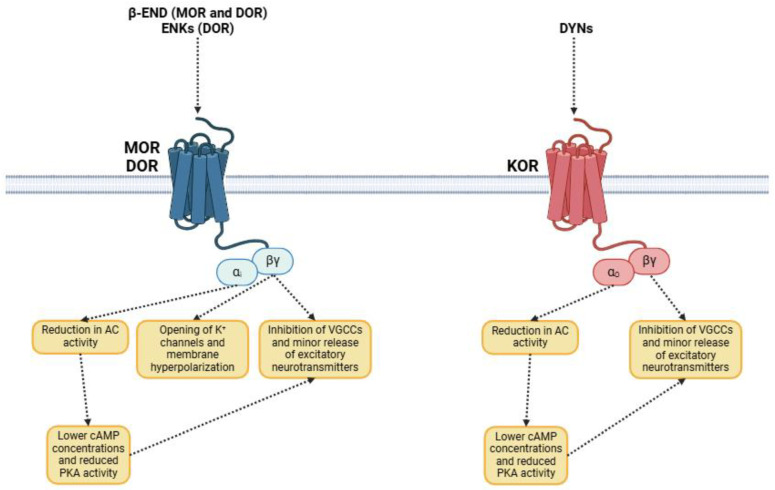
Signaling cascade initiated by the activation of the opioid receptors. Abbreviations: β-END (β-endorphin); ENKs (enkephalins); DYNs (dynorphins); AC (adenylyl cyclase); MOR (μ-opioid receptor); DOR (δ-opioid receptor); KOR (κ-opioid receptor); cAMP (cyclic adenosine monophosphate); PKA (protein kinase A); VGCCs (voltage-gated calcium channels).

Opioid receptors (Figure 1) belong to the family of G protein-coupled receptors, the so-called GPCRs [29]. The basic structure of GPCRs consists of a polypeptide chain that crosses the cell membrane seven times. GPCRs have an extracellular N-terminal domain of variable length and an intracellular C-terminal domain that interacts with heterotrimeric G-proteins [30,31]. The objective of G-protein signaling, along with adenylyl cyclase (AC), the enzyme responsible for the production of the second messenger cAMP, is phospholipase C (PLC), which mediates the strong formation of the second messengers inositol triphosphate (IP3) and diacylglycerol (DAG), and several ion channels, including those involved in calcium and potassium transport [32]. Opioid receptors represent a complex group of GPCRs involved in several biological functions [29]: modulation of painful sensation, reward processing, and many physiological functions within organisms. These receptors are subdivided into three main subtypes (μ -MOR-, δ -DOR-, and κ -KOR-), each with distinct pharmacological profiles and distribution patterns in the CNS, PNS, and other tissues [33].

#### 2.1.1. μ-Opioid Receptors (MORs)

MORs are a subclass of GPCRs that cause supraspinal analgesia, respiratory depression, euphoria, sedation, reduced gastrointestinal motility, and dependence (Table 2) [34]. The human *OPRM1* gene, located on chromosome 6 (6q25.2), comprises a 236.37 kb sequence with 19 exons and 18 introns [35]. This gene is highly expressed in the CNS but is also present within the PNS and other tissues [36,37]. Several receptor isoforms (up to 34) are formed by alternative splicing through transcription of different mRNA variants, resulting in distinct protein isoforms that have unique properties and tissue distribution patterns [37]. Activation of MORs primarily involves interaction with an inhibitory G protein (Gα_i_), which inhibits the AC and reduces intracellular cAMP levels [38]. This triggers a downstream signaling cascade, with reduced neuronal excitability and neurotransmitter release and changes in synaptic plasticity [38]. The Gβγ subunits are involved in the opening of G protein-coupled inward rectifying potassium channels (GIRKs) and the inhibition of voltage-gated calcium channels (VGCCs), resulting in hyperpolarization of the plasmatic membrane, a further decrease in neurotransmitter release, and a potentiation of the analgesic effect [39].

In contrast, chronic exposure to exogenous opioids (such as morphine) leads to desensitization of the MOR, downregulation, and altered receptor trafficking, processes associated with tolerance and dependence [40]. Polymorphisms in the *OPRM1* gene, such as the extensively studied single nucleotide polymorphisms (SNPs; rs1799971), modify receptor expression and/or binding affinity, resulting in individual differences in response to many opioid medications [41]. Furthermore, MORs are implicated in the modulation of some types of stress, emotional regulation, and reward pathways, making them a crucial element in understanding addiction and psychiatric disorders [42,43,44]. In addition to pain and reward, these receptors are known to modulate gastrointestinal motility and respiration [45,46].

#### 2.1.2. δ-Opioid Receptors (DORs)

The DOR, which is encoded by the *OPRD1* gene, is a member of GPCRs and is also very important in the regulation of emotional responses as well as pain intensity (Table 2) [47,48,49]. The human *OPRD1* gene is reported to be a 59.1 kb sequence located on chromosome 1 (1p35.3), comprising six exons and five introns [35]. This gene is highly expressed in the CNS, particularly in those areas implicated in the processing of emotions, such as the amygdala, nucleus accumbens, and several cortical areas [50]. DORs may participate in one of the cardioprotective mechanisms for the protection of cardiac tissues against an ischemic insult through a reduction in the oxidative stress and preservation of mitochondrial function [51]. In addition, DORs are found in peripheral tissues, including immune cells, and may play a key role in modulating inflammatory responses [50,52,53].

DORs preferentially couple with the inhibitory Gα_i_ protein, which blocks AC and, thereby, diminish the levels of the intracellular messenger cAMP [50]. This pathway reduces neuronal excitability and neurotransmitter release, like the effect resulting from the activation of MORs [50]. As in MORs, the Gβγ proteins promote the opening of potassium channels and the inhibition of VGCCs, hyperpolarizing the plasma membrane and therefore diminishing neurotransmitter release [54]. Simulation of DORs modulates the activity of mitogen-activated protein kinase (MAPK)/extracellular signal-regulated kinase (ERK), subsequently impacting cell function, survival, and synaptic plasticity [50]. Alternative splicing results in a variety of DOR isoforms (up to six) that have many functional properties. These involve changes in ligand affinity and efficacy, as well as a preference for G protein coupling, which forms the basis for tissue-specific functions and variable tissue responses to opioid ligands [35]. Indeed, the human *OPRD1* gene has been linked by polymorphisms to individual variations in pain perception and susceptibility to addiction and even psychiatric disorders, demonstrating that the role of this receptor extends far beyond nociception [55,56]. Some SNPs have been associated with functional or expression changes in the receptor that may be relevant to either susceptibility to opioid addiction or response to opioid-based treatments [48].

#### 2.1.3. κ-Opioid Receptors (KORs)

The KOR, which belongs to the GPCR family, is encoded by the *OPRK1* gene on human chromosome 8 (8q11.23) [57]. The *OPRK1* gene consists of a 25.91 kb long sequence comprising nine exons and eight introns [35]. It is widely expressed in the CNS (e.g., prefrontal cortex, substantia nigra, raphe nuclei, or hippocampus), PNS, and peripheral tissues (e.g., immune cells), indicating that KORs play an important role in the modulation of pain as well as regulating affective and emotional states [58]. Like other opioid receptors, the KOR has characteristic seven transmembrane-spanning motifs with an extracellular N-terminus and an intracellular C-terminus linked to the Gα_o_ protein [58]. After ligand interaction, activation of KOR inhibits AC, which, consequently, reduces intracellular cAMP levels. In addition, this interaction prompts the action of Gβγ subunits, which, in turn, inhibit VGCCs, reducing the release of excitatory neurotransmitters and lowering neuronal excitability, thereby supporting analgesic responses [59,60]. Pain modulation is one of the major functions of KORs in the CNS, particularly through supraspinal analgesia [61]. Activation of KORs (expressed in the spinal dorsal horn) can block the release of excitatory neurotransmitters, including glutamate and substance P, which in turn attenuates nociceptive signaling [62].

In contrast to MORs, KORs are not involved in reward pathways and elicit aversive effects, which not only limit their abuse potential (Table 2) [63]. Dysphoria and hallucinations can also be induced by the activation of KORs [64]. In stressful situations, the activation of KORs mediates dysphoric and anxiety-inducing effects, which are primarily characterized by a significant reduction in dopamine release in brain regions associated with reward. This effect contrasts with that of MOR activation, which increases dopamine levels and is associated with feelings of pleasure [65].

Environmental and genetic factors regulate the expression of the *OPRK1* gene. Numerous SNPs have been associated with variability in both KOR function and expression. These SNPs have been associated with individual differences in stress resistance, addiction susceptibility, and response to opioid-based treatments, highlighting the major influence of KORs on behavior [66]. Alternative splicing of OPRK1 transcripts leads to the generation of multiple receptor isoforms. These isoforms may display diverse signaling properties and tissue-specific functions, allowing them to activate different intracellular pathways or to interact variably with their corresponding ligands [35].

### 2.2. Opioid Peptides

Endogenous opioids are neurotransmitters and neuromodulators that have a significant impact on the nervous system, particularly on pain regulation and reward mechanisms, by modulating numerous electrical properties of neurons and, thereby, reducing their responsiveness to many stimuli [67]. Like other peptides, endogenous opioids have an atypical synthetic pathway that begins from large precursor proteins that are further cleaved and processed into biologically active forms [68]. Proopiomelanocortin (POMC), proenkephalin (PENK), and prodynorphin (PDYN) are three precursor proteins that lead to three major classes of opioid peptides: β-END, ENK, and DYN, respectively [68].

Each endogenous opioid peptide interacts with different opioid receptors in the CNS and peripheral tissues and produces many physiological and pharmacological effects [33]. Common to opioid peptides is a conserved amino-terminal sequence, which is crucial for binding to the opioid receptors [69]. This motif is found in several opioid peptides, a phenomenon which emphasizes its functional importance for the interaction and activation of opioid receptors.

#### 2.2.1. Biosynthesis

The biosynthesis of opioid peptides is a very complicated and highly regulated process that occurs mainly within neurons and endocrine cells. Endogenous opioid peptides have a typical amino-terminal sequence (commonly known as “opioid motif”; Tyr-Gly-Gly-Phe) which is important for their biological activity [69]. This process begins with the transcription of several genes encoding precursor proteins, followed by their subsequent translation. This is accompanied by other cotranslational events, which include the cleavage of the signal peptide [70,71]. The precursor proteins are then secreted by transport through the Golgi apparatus, where they undergo additional processing via post-translational modifications that include the addition of carbohydrate and phosphate groups [70,71]. The processed precursor proteins are, subsequently, packaged into secretory vesicles, where they are further cleaved by specific enzymes to form active opioid peptides [71].

**Figure 2:**
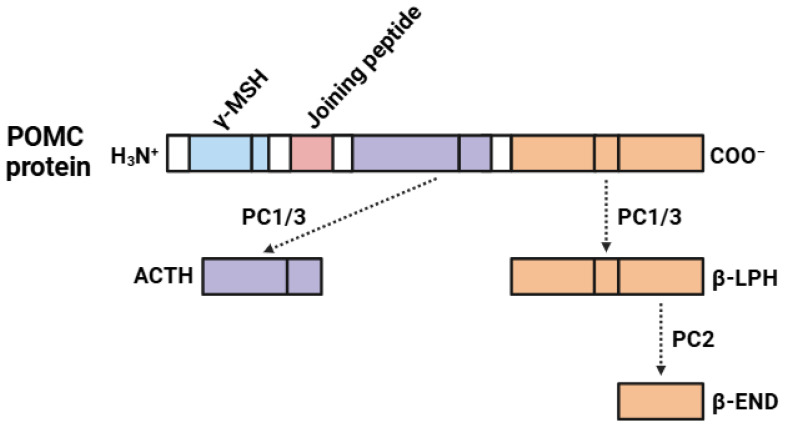
Biosynthetic pathway of the β-endorphin (β-END). Abbreviations: γ-MSH (γ-melanocyte-stimulating hormone); ACTH (adrenocorticotropic hormone); POMC (proopiomelanocortin); PC1/3 (prohormone convertase 1/3); β-LPH (β-lipotropin); PC2 (prohormone convertase 2); β-END (β-endorphin). The colors indicate: light blue (γ-MSH), pink (joining peptide), dark blue (ACTH), and orange (β-LPH and β-END).

The biosynthesis of β-END (31 amino acids; 3.5 kDa) is a regulated process involving a complex sequence of molecular events (Figure 2), generally in the pituitary gland and in several neurons of the CNS. The precursor protein for the biosynthesis of β-END is POMC [72]. POMC (241 amino acids; 31 kDa) is a protein involved in the biosynthesis of several biologically active peptides, like β-END. As a result, POMC undergoes post-translational modifications and proteolytic cleavages to produce numerous bioactive peptides [73,74]. The first step in processing involves the cleavage of POMC by prohormone convertases (PC1/3 and PC2), enzymes that recognize specific sequences within the POMC, commonly Lys-Arg or Arg-Arg [75]. PC1/3 cleaves POMC into adrenocorticotropic hormone (ACTH) and β-lipotropin (β-LPH) [75]. ACTH is a peptide of 39 amino acids that stimulates cortisol production in the adrenal glands during the stress response [76]. In contrast, β-LPH is larger and consists of 93 amino acids [77]. Further processing of β-LPH by PC2 generates smaller peptides, including γ-lipotropin (γ-LPH) and β-END [78]. In addition to β-END, the cleavage of POMC produces a cluster of other bioactive peptides, including peptides such as α-melanocyte-stimulating hormone (α-MSH) and γ-melanocyte-stimulating hormone (γ-MSH).

**Figure 3:**
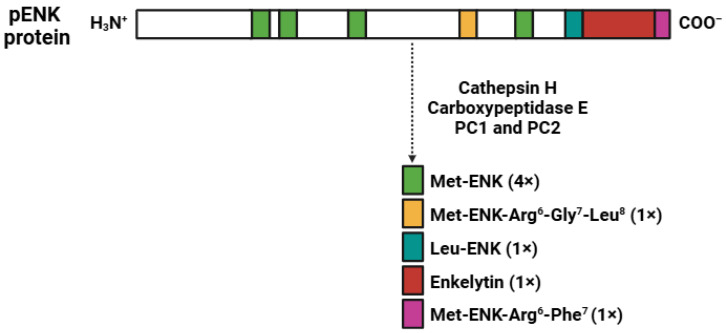
Biosynthetic pathway of the enkephalins (ENKs). Abbreviations: pENK (proenkephalin A); Met-ENK (met-enkpehalin); Leu-ENK (leu-enkephalin); PC1 and PC2 (prohormone convertase 1 and 2). The colors indicate: green (Met-ENK), yellow (Met-ENK-Arg^6^-Gly^7^-Leu^8^), blue (Leu-ENK), red (enkelytin), and pink (Met-ENK-Arg^6^-Phe^7^).

The biosynthesis of ENKs (five amino acids; approximately 0.6 kDa) follows a complex pathway (Figure 3) that begins with the production of a larger precursor molecule, proenkephalin A (pENK). This precursor (243 amino acids; 5.3 kDa) contains four copies of Met-ENK and one copy of Leu-ENK, Met-ENK-Arg^6^-Phe^7^, Met-ENK-Arg^6^-Gly^7^-Leu^8^, and enkelytin [24]. The conversion of pENK into functional opioid peptides requires the ynchronized action of various peptidases, including PC1 and PC2, carboxypeptidase E, and cathepsin H [79,80,81].

**Figure 4:**
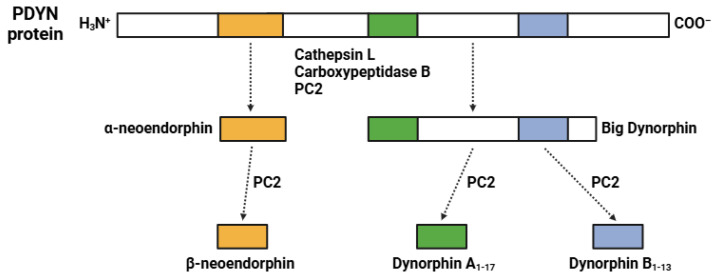
Biosynthetic pathway of the dynorphins (DYNs). Abbreviations: PDYN (prodynorphin); PC2 (prohormone convertase 2). The colors indicate: yellow (α-neoendorphin and β-neoendorphin), green (dynorphin A_1-17_), and blue (dynorphin B_1-13_).

Finally, the biosynthesis of DYNs (Figure 4) is very complex and involves the production of a precursor protein called prodynorphin, pDYN (254 amino acids; 28.4 kDa). Numerous non-selective proteases (such as cathepsin L, PC2, and carboxypeptidase B) are involved in the processing of pDYN [82,83,84]. In particular, the enzyme PC2 cleaves pDYN in presynaptic vesicles into active peptides, which include dynorphin A1-17 (17 amino acids; 2.15 kDa), dynorphin B1-13 (13 amino acids; 1.57 kDa), α-neoendorphin (10 amino acids; 1.3 kDa), and β-neoendorphin (9 amino acids; 3.47 kDa) [83,84]. DYNs are synthesized in response to sustained neuronal activation that triggers the processing of pDYN by PC2 in synaptic vesicles, which are then released into the synaptic cleft [83].

#### 2.2.2. Distribution

The distribution and processing of opioid peptides (and their precursor mRNAs) has been studied primarily in several brain regions, with more recent studies extending this focus to other cells. Larger databases, including the Human Protein Atlas and mouse brain studies, show that mRNAs encoding precursors of opioid peptides (POMC, PENK, and PDYN) are not exclusively expressed in the SNC, but are also highly expressed in peripheral tissues such as the adrenal gland, testis, immune cells, and heart, suggesting a variety of physiological functions [23,69,85,86].

The mRNA expression of POMC has a specific distribution profile in human tissues. The anterior pituitary is considered the most important tissue with high POMC mRNA expression [87]. Other areas where POMC mRNA has been detected include the hypothalamus [88], skin [89], and various types of immune cells (e.g., lymphocytes) [90].

PENK mRNA is found in a variety of tissues. The *PENK* gene is highly expressed in various parts of the CNS and PNS, such as the hippocampal formation, amygdala, basal ganglia, midbrain, spinal cord, and cerebral cortex [24]. Moreover, PENK mRNA is expressed in numerous endocrine glands, including adrenal medulla, thyroid, parathyroid, pituitary, and pancreas [91,92,93,94]. Surprisingly, PENK mRNA is also expressed in some non-neuronal cells, including keratinocytes and melanocytes, among others [95,96]. In the kidney, PENK mRNA has been detected in the proximal tubule [97]. Other zones where PENK mRNA has been found include several immune cells (e.g., CD4^+^ T lymphocytes) and reproductive organs [24,98,99,100,101].

Finally, the distribution of PDYN mRNA throughout the CNS has been investigated in detail. High expression of PDYN has been found in the striatum, including the nucleus accumbens and putamen [102,103,104]. An important structure of the basal ganglia, the substantia nigra, has substantial amounts of PDYN mRNA, suggesting a possible role in dopaminergic signaling [105]. Other areas of the CNS include the hypothalamus [106], hippocampus [107], amygdala [108], periaqueductal gray [109], and spinal cord [110]. In peripheral tissues, the highest concentrations of mRNA DYN have been detected in the stomach and upper intestine [111,112]. Finally, DYNs have also been discovered in immune cells such as neutrophils [113].

## 3. Fibromyalgia: An Overview

Fibromyalgia is a chronic disease characterized by widespread musculoskeletal pain, although it is linked to symptoms like fatigue, sleep disturbances, and anxiety [1,2,3,4,5,6,7,8,9,10,11,12]. This condition affects approximately 1 to 5% of the general population, depending on the diagnostic criteria used [114]. Although the etiology of fibromyalgia has not yet been clarified, new research findings suggest a connection with disorders of pain regulation [115]. Fibromyalgia patients show increased pain sensitivity and altered pain processing as well as changes in the mechanisms responsible for pain modulation [18,19,20,21].

The management of fibromyalgia is based on a patient-centered approach [116]. This approach adapts numerous treatments to the individual needs of patients, while offering support in managing the psychological and emotional effects of the disease. Fibromyalgia management requires continuous adjustments and a cooperative relationship between the patient and their treatment team [116].

### 3.1. Signs and Symptoms

Fibromyalgia is a disorder characterized by a variety of symptoms that complicate the diagnostic process. An accurate diagnosis requires a comprehensive evaluation of the medical history, combined with a complete physical examination [117]. Fibromyalgia is characterized by persistent, widespread musculoskeletal pain, usually affecting both sides of the body. Predominantly, this pain involves the following regions: lumbar, gluteal, cervical, scapular, and dorsal paravertebral, all of which worsen the pain scores in the cervicoscapular and lumbar-pelvic regions [16,118,119]. Fibromyalgia is associated with significant posterior axial pain, as well as headaches that are mainly temporal, retro-orbital, and frontal. Although peripheral pain in the limbs is very rare, it has been observed in the feet, arms, and, often, in the knees, elbows, and hands [16,118,119]. On the other hand, this interplay of symptoms requires a careful diagnostic approach, as an incomplete understanding can lead to an incorrect treatment of the condition.

Fibromyalgia pain exhibits considerable variation over time, influenced by a variety of factors: temporal context, seasonal variation, and level of physical activity [120]. Inactivity becomes a key factor that exacerbates symptoms and creates a vicious cycle in which pain reduces activity levels, a phenomenon which, in turn, exacerbates the intensity of pain [120]. In addition, stress, humidity, and low temperatures increase the sensation of pain [121].

Patients with fibromyalgia often suffer from severe fatigue, regardless of the amount or quality of sleep [122]. Sleep disturbances are prevalent among these individuals, who often encounter difficulties in falling asleep, maintaining sleep, or attaining restorative slumber [123]. In addition, many people with fibromyalgia suffer from cognitive impairment, commonly referred to as “fibro fog”, which encompasses difficulty concentrating, memory impairment, and problems performing mental tasks [124]. Those symptoms can interfere significantly with daily functioning, underscoring the necessity for treatments that address both the musculoskeletal and systemic manifestations of the condition.

### 3.2. Pathophysiology

The exact pathophysiological mechanisms underlying fibromyalgia are still not fully understood. However, this disorder is thought to be caused by many factors, including impaired cortical processing, reduced inhibitory pain modulation, and molecular changes within pain pathways [118,119]. It is evident that those individuals suffering from fibromyalgia exhibit increased sensitivity to various stimuli (mechanical and ischemic stresses as well as thermal extremes such as heat and cold) [125].

The pathophysiology associated with this increased sensitivity to pain has been extensively studied and is thought to be related to CNS dysregulation manifesting at both the cerebral cortex and spinal cord levels [126]. Among the central alterations found in fibromyalgia are irregularities in monoaminergic neurotransmission, leading to increased levels of some excitatory neurotransmitters (known as algesic mediators) such as glutamate and substance P [127,128]. This is exacerbated by reduced levels of serotonin and noradrenaline within the spinal cord, principally affecting the descending antinociceptive pathways [129,130]. The anterior cingulate cortex, rostral ventromedial medulla, and periaqueductal gray are key components of the descending pain modulation pathway. This system plays a pivotal role in regulating pain, primarily through the release of endogenous opioids at the spinal level via serotonergic and noradrenergic pathways [131,132].

Additionally, some morphological changes in the CNS contributing to central sensitization have been found in fibromyalgia patients. Notable gray matter volume reductions have been detected in voxel-based morphometry studies, especially within the anterior cingulate cortex and prefrontal cortex [133]. This is further supported by functional magnetic resonance imaging (fMRI) studies, which demonstrate a heightened activity across the pain matrix in fibromyalgia patients, indicating an increased sensitivity to nociceptive stimuli [134]. The descending pain-modulating system in these patients also demonstrates a reduction in functional connectivity which may be part of the pathophysiology leading to the amplification of pain signals [135]. Neuroimaging studies have indicated changes in the connectivity of the pain network while at rest, suggesting a state of persistent central sensitization [133]. Indeed, at the level of the spinal cord, sensitization has been attributed to increased excitability of the dorsal horn neurons that contributes to increased pain signal transmission [136]. These modifications in the CNS support allodynia, hyperalgesia, and sensory hyperresponsiveness in fibromyalgia [137].

On the other hand, when Aδ and C fibers form synaptic connections within the dorsal horn, they release a triad of neurotransmitters (glutamate, calcitonin gene-related peptide –CGRP–, and substance P), thereby initiating the cascade of pain signal transmission [138]. In the spinal dorsal horn, opioid peptides act on interneurons at presynaptic and postsynaptic sites. Upon activation of their receptors, hyperpolarization occurs in ascending fibers and the presynaptic release of glutamate and substance P is blocked, decreasing the transmission of pain signals to the brain [139]. Chronic stimulation of C fibers, a phenomenon which is observed in numerous cases of fibromyalgia, may lead to the apoptotic loss of inhibitory opioid and GABAergic interneurons; however, this phenomenon is accompanied by a continuous release of glutamate and substance P onto the second-order neurons, alongside diminished central levels of opioid peptides and serotonin [139,140].

Peripheral mechanisms are fundamentally pivotal to the initiation and advancement of fibromyalgia. Recent investigations have demonstrated the role of small fiber neuropathy, characterized by irregularities in both Aδ and C fibers [141]. In this context, inflammatory mediators (e.g., substance P and CGRP) are crucial in the sensitization of transient potential vanilloid receptors (e.g., TRPV1), acid-sensing ion channel receptors (ASICs), and purinergic receptors (e.g., P2X3) [141]. However, these mediators interact with receptors placed in inflammatory cells, which include neutrophils and basophils [142]. This interaction triggers the release of numerous pro-inflammatory mediators, which encompass cytokines and chemokines [143,144]. Furthermore, one recent investigation has shown alterations in the density and morphology of peripheral nerve fibers in fibromyalgia patients, possibly leading to modified pain processing [145].

This dynamic interplay between peripheral and central mechanisms constructs a complex pain amplification framework. Essentially, this system entails an upregulation of pro-inflammatory cytokines (like IL-6 and TNF-α) and a downregulation of anti-inflammatory cytokines (like IL-10) [146]. The activated peripheral nociceptors may release the neuropeptides (substance P and CGRP) into the surrounding tissue, thus promoting local inflammation and further sensitization of the adjacent nociceptors [147]. This neurogenic inflammation can secondarily result in the central sensitization process due to the delivered sustained nociceptive input to the spinal cord and higher brain centers [148].

Numerous factors seemingly contribute to the pathophysiology of fibromyalgia, including genetic predisposition, immune system functionality, and oxidative stress, among others. Genetic predisposition is particularly significant, as research has pinpointed specific genetic markers correlated with a high risk of fibromyalgia onset [149]. This condition is projected to possess a heritability rate of roughly 50%, thereby emphasizing the critical influence of genetic elements [150]. Variations in genes linked to pain perception, neurotransmitter modulation, and immune response have been implicated (e.g., *SLC64A4*, *TRPV2*, *MYT1L*, and *NRXN3*) [150,151]. Identifying these genes provides a basis for establishing a foundation for understanding the genetic aspects of fibromyalgia. However, it is essential to consider the interaction between environmental and genetic factors [150]. Environmental influences, such as physical trauma, infections, or a stressful life, can serve as catalysts in individuals genetically predisposed, thereby facilitating the development of fibromyalgia [149].

Emerging evidence suggests that dysregulation of the immune system may play a significant role in the etiology of fibromyalgia. A recent investigation [152] examined the characteristics of monocytes and the responses of peripheral blood mononuclear cells (PBMCs), revealing significant variations in the percentages of monocytic cells among individuals diagnosed with fibromyalgia, particularly under unstimulated conditions. This research delineated three pivotal findings [152]: the first observation highlighted differences in monocytic cell percentages among fibromyalgia patients, especially when considering baseline conditions, the second discovery indicated a diminished percentage of CD19^+^ B cells in those patients with fibromyalgia, and, finally, the third finding revealed reduced stimulation indices of IFN-γ within the fibromyalgia cohort. Additional research indicates that patients with fibromyalgia exhibit alterations of the innate and adaptative immune system. Mast cells in these individuals produce elevated levels of pro-inflammatory cytokines (such as IL-6, IL-17, and TGF-β), while neutrophils release IL-6, IL-8, and TNF-α. Microglial cells demonstrate high levels of substance P expression, with an imbalance in the M1/M2 macrophage ratio that favors the pro-inflammatory M1 phenotype [153]. Moreover, the involvement of Th1 and Th17 T cell subsets is suggested, along with a possible autoimmune component involving autoreactive T cells [154].

Furthermore, mitochondrial dysfunction (characterized by an excessive biosynthesis of reactive oxygen species, –ROS–) has been linked to fibromyalgia, suggesting that disrupted energy metabolism might be implicated in this condition [155]. In this regard, oxidative stress can increase pain sensitivity in fibromyalgia patients by activating nociceptors and modifying pain pathways [155]. In patients diagnosed with fibromyalgia, there exists a notable reduction in the activity of antioxidant enzymes, including catalase and superoxide dismutase (SOD). This decline might indicate a strong impairment of antioxidant defenses [156,157]. Although the implications are very disconcerting, further investigation is warranted to elucidate the mechanisms involved, because understanding these pathways may lead to improved therapeutic strategies [155].

## 4. Relation Between the Endogenous Opioid System and Fibromyalgia

The role of the endogenous opioid system in the context of fibromyalgia is presumably critical, given its influence on both the pain experience and the progression of this disease [22]. Fibromyalgia individuals may exhibit a dysregulation within this system which results in a heightened sensitivity to pain [22]. This dysfunction in opioid signaling constitutes a part of the dysregulation in the systems that modulate pain and underlines the complexity of fibromyalgia as a disorder that involves peripheral and central mechanisms [133,141]. Some authors suggest that impairments within the endogenous opioid system, such as those proposed here, may, over time, lead to the hyperactivation of pain pathways, and, therefore, transform normal stimuli into painful or heightened responses toward typical pain stimuli (allodynia and hyperalgesia, respectively) [137].

The dysregulation of the opioid system in fibromyalgia is closely associated with a variety of neurobiological dysfunctions, including imbalances in major neurotransmitter systems, especially serotonin and noradrenaline [158]. These abnormalities extend to the hypothalamic–pituitary–adrenal (HPA) axis, which plays an essential role in the complex pathophysiology of fibromyalgia [159]. Dysfunctional signaling within the HPA axis can alter the levels of cortisol and other stress hormones, increasing the sensitivity to pain [160].

The HPA axis and the opioid system are closely interconnected. The regulation of β-END release is intricately dictated by the activity of the HPA axis [161]. In a dysfunctional state, observed in fibromyalgia, the equilibrium that exists between endogenous opioid biosynthesis and receptor sensitivity may become severely disrupted [134]. This disturbance results in a substantial decrease in the efficacy of the pain-relief mechanisms, as the balance for their performance is clearly disrupted [22]. Moreover, the impairment of the HPA axis begins a neuroinflammatory process [162,163], increasing the sensitization of nociceptive pathways and promoting central sensitization, a mechanism involved in the enhanced pain experience in fibromyalgia patients [125,133,164,165].

Several investigations have shown that fibromyalgia patients show high concentrations of endogenous opioids within their cerebrospinal fluid (like Met-ENK-Arg^6^-Phe^7^), thereby signifying an upregulation of this system in reaction to this condition [166]. However, this augmentation in endogenous opioids is paradoxically associated with a strong reduction in MORs in key pain-regulating regions of the SNC, such as the thalamus, insula, cingulate cortex, amygdala, and nucleus accumbens [167,168]. This reduced availability of MORs may result from chronic overstimulation and the subsequent downregulation of these receptors. Persistent pain (key symptom derived from fibromyalgia) often creates a continuous demand for endogenous opioids, leading to the desensitization of the receptors over time [40,169]. In fibromyalgia, the overstimulation of MORs (caused by increased concentrations of opioid peptides) has the potential to disrupt the equilibrium between excitatory and inhibitory neurotransmitters within the CNS. In the context of fibromyalgia, there is an excess of excitatory neurotransmitters (e.g., glutamate and substance P) [127,128] and a misfunction of the opioid receptors, leading to an enhanced perception of pain [126]. The molecular mechanisms underlying the dysregulation may include the phosphorylation and subsequent internalization of CNS MORs, which may occur due to the sustained release of endogenous opioids [170]. This molecular mechanism elucidates why traditional opioid therapies often fall short of delivering sufficient pain relief for individuals afflicted with fibromyalgia, because the receptors responsible for mediating the analgesic effects of these opioids become less accessible [169,171].

On the other hand, neuroinflammation and oxidative stress play essential roles in the pathology of fibromyalgia because they affect both the opioid system and glial cells. Some studies have shown elevated levels of oxidative stress markers in patients with fibromyalgia, suggesting that mitochondrial dysfunction and subsequent oxidative damage significantly contribute to the etiology of this condition [172]. Glial cells are recognized as highly reactive and immunocompetent entities within the CNS. These cells are highly activated in response to various stimuli, such as inflammation, tissue damage, and various forms of stress [173]. In fibromyalgia, glial activation triggers the release of pro-inflammatory cytokines, disrupts balance neurotransmitter, and increases neuronal excitability, all of which contribute to the amplification of pain sensations [174,175]. The interaction among neuroinflammation, oxidative stress, and the opioid system (in fibromyalgia) is highly complex. Moreover, activated glial cells release pro-inflammatory cytokines which exacerbate pain signals and interfere with opioid signaling pathways, thereby reducing the capacity to manage pain effectively [176,177,178].

The interplay between endogenous opioid peptides and sensitized nociceptors in the context of fibromyalgia remains insufficiently understood; however, it is probable that the altered opioid system is inadequate for effectively mitigating the intensified activity of nociceptors [179]. In this condition, the process of peripheral sensitization is linked to neurogenic inflammation, including the release of certain pro-inflammatory mediators such as substance P, CGRP, and NGF [141]. These substances promote not only the sensitization of nociceptors, but also play a key role in maintaining pain [146]. Opioid peptides have been demonstrated to modulate the production and release of these pro-inflammatory mediators, indicating that peripheral opioid system dysfunction might play an essential role in maintaining neurogenic inflammation and, consequently, the peripheral sensitization observed in fibromyalgia patients [125].

Additionally, the complex interactions between the endogenous opioid system and the TRPV1 channels, coupled with the increased likelihood of opioid tolerance, present additional challenges in developing effective treatment strategies [180]. Strong association of the TRPV1 channels is seen in the potentiation of opioid analgesia during acute inflammatory conditions [181]; however, dysregulation of these channels is a critical factor in the transition from acute to chronic pain that is usually observed in fibromyalgia [181,182]. Altered TRPV1 channel activity may sustain nociceptive signaling and amplify sensitization across pain pathways, resulting in pain that emerges as increasingly chronic and resistant to conventional opioid treatments over time [183].

Recent findings regarding immune cell involvement (mainly neutrophils) have elucidated an unanticipated role in pain modulation within the framework of fibromyalgia, suggesting that these immune cells might influence pain perception through the atypical expression of opioid peptides [184]. Other immune cells, especially B cells, express MORs and they have emerged as a potential biomarker for chronic pain conditions such as fibromyalgia [185]. These findings reveal a complex mechanism involving both the immune and nervous systems in pain modulation. The relationship between these physiological systems is multifaceted, playing a crucial role in how pain is processed and regulated, with significant implications for developing effective treatments [186,187,188].

## 5. Opioid-Based Pharmacological Therapies Against Fibromyalgia

A substantial body of research has highlighted a range of pharmacological interventions used as supportive therapies in the management of fibromyalgia. The predominant objective of these pharmacological agents is to improve sleep quality, mitigate depressive and anxiety symptoms, and/or reduce fatigue [189].

With respect to the administration of opioids (Table 3), some clinical trials and the guidelines established by EULAR (European Alliance of Associations for Rheumatology) do not endorse their use in the treatment of fibromyalgia, and tramadol (characterized as a MOR agonist with SNRI activity) is recommended [190,191]. Furthermore, tramadol can serve as a second-line therapeutic intervention for those patients that show severe signs and symptoms [190,192]. Conversely, a particular investigation revealed that tapentadol extended–release was linked to analgesic effects and an enhancement in the quality of life among individuals suffering with fibromyalgia [193]. Common side effects for both medications include nausea, constipation, dizziness, and somnolence. Other adverse effects include respiratory depression, cardiovascular problems (such as tachycardia), seizures, risk of dependence, and withdrawal symptoms upon abrupt discontinuation [194].

Low-dose naltrexone (LDN) therapy has emerged as a promising approach to the treatment of patients with fibromyalgia, offering potential advantages characterized by reduced side effects [195]. Naltrexone, a non-selective opioid antagonist, is thought to act through many mechanisms, influencing several biological processes that are involved in pain regulation and immune function. These include immune response modulation [196], increased opioid peptide biosynthesis [197], and immune system stabilization [198]. The safety profile of LDN is generally favorable, where most adverse effects tend to be mild and transient. These usually include insomnia, headaches, and nausea [195].

On the other hand, a survey revealed that 75% of patients found hydrocodone (combined with acetaminophen) to be very effective, while 67% reported similar efficacy with oxycodone (combined with acetaminophen) [199]. Fibromyalgia has been strongly linked with preoperative opioid use, including hydrocodone; however, data from randomized controlled trials on the benefits or risks of oxycodone for fibromyalgia are strongly limited [200].

Codeine (weak opioid agonist) has been evaluated in several comparative studies, but patients in the codeine–acetaminophen group reported higher rates of drowsiness and constipation, whereas those in the tramadol–acetaminophen group experienced more headaches [201]. Both treatments showed comparable effectiveness for managing chronic pain; this is important, as it underscores the complexities involved in implementing pain management strategies in clinical settings.

Finally, fentanyl (potent opioid agonist) mainly acts on MORs and has been demonstrated to relieve pain in fibromyalgia patients [202]. However, its utilization carries a high risk of overdose. This concerning trend highlights the urgent need for further research to elucidate the physiological mechanisms of a fentanyl overdose, because effective interventions aimed at reducing mortality must be developed [203].

## 6. Other Fibromyalgia Management Strategies

Although opioids are occasionally prescribed for pain relief, their use in the context of fibromyalgia is a topic of ongoing discussion due to concerns about long-term efficacy and potential side effects. This has led to an emphasis on alternative management strategies, such as cognitive–behavioral therapy, moderate physical exercise, and mindfulness practices; however, non-opioid pharmacological treatments are also considered crucial in providing pain relief and supporting an improved lifestyle for patients.

### 6.1. Pharmacological Strategies

A considerable body of investigations has elucidated numerous pharmacological interventions employed as supportive therapies in the management of fibromyalgia. These interventions encompass antidepressants, monoamine reuptake inhibitors, serotonin, and dopamine receptor antagonists, gabapentinoids, and cannabinoids, as well as others [204]. The main objective of these pharmacological agents is to improve sleep quality, mitigate depressive and anxiety symptoms, and/or reduce fatigue. The pharmacological agents utilized in fibromyalgia are delineated as follows.

#### 6.1.1. Tricyclic Antidepressants (TCAs)

These drugs are thought to exert their action on pain perception by modulation of serotonin and noradrenaline neurotransmission, together with the effects on potassium channels and NMDA receptors [205]. The strongest evidence for fibromyalgia management emphasizes the effectiveness of amitriptyline and nortriptyline [206,207]. Research indicates that amitriptyline can achieve a 30% reduction in FIQ scores [207]. Doxepin, another TCA, has manifested beneficial outcomes in individuals suffering from fibromyalgia [208]. While TCAs can be highly effective, their employment might be limited by side effects such as dry mouth, constipation, and daytime drowsiness [209].

#### 6.1.2. Serotonin–Noradrenaline Reuptake Inhibitors (SNRIs)

The main SNRIs used in fibromyalgia management are duloxetine and milnacipran. Both pharmacological agents have demonstrated efficacy in alleviating a variety of symptoms associated with this complex condition [210,211]. These drugs increase the concentration of serotonin and noradrenaline within the CNS, a phenomenon which is thought to play an essential role in the modulation of pain as well as in the expression of other significant symptoms of fibromyalgia, such as depression [212]. These drugs are not devoid of side effects; nausea and drowsiness are the most frequent complaints [213]. However, although this may be a challenge, the possible benefits of SNRIs in managing fibromyalgia symptoms often outweigh these limitations, as they can improve the quality of life for many patients [213].

#### 6.1.3. Selective Noradrenaline Reuptake Inhibitors (NRIs)

The justification for using NRIs in the management of patients with fibromyalgia is an appreciation that noradrenergic dysfunction participates in its pathophysiology, especially in relation to pain modulation and cognitive function [214]. Reboxetine has demonstrated significant potential in alleviating the fibromyalgia pain-related symptoms [215]. Esreboxetine, the active enantiomer of reboxetine, has also been shown to be effective in improving FIQ scores [216]. One case report highlights the efficacy of atomoxetine at treating an individual with fibromyalgia [217]. Adverse effects marked for NRIs include headache, dry mouth, abdominal pain, nausea, and insomnia [213].

#### 6.1.4. Selective Serotonin Reuptake Inhibitors (SSRIs)

SSRIs increase serotonin concentrations significantly within the CNS, a phenomenon which is theorized to be important for pain and mood modulation [218]. SSRIs include citalopram, escitalopram, fluoxetine, paroxetine, and sertraline [219,220,221,222,223]. As shown in the meta-analysis conducted by Häuser et al. [224], SSRIs may provide modest improvements in pain, depressive symptoms, and overall quality of life, but their impact on sleep disorders appears to be minimal. This might indicate the complex position SSRIs hold in symptom management for fibromyalgia, but further research is certainly warranted to fully elaborate their efficacy across disease manifestations. Adverse effects reported include nausea, dyspepsia, anorexia, dizziness, blurring of vision, dry mouth, sweating, sleep disturbance, headache, and sexual dysfunction [225].

#### 6.1.5. Serotonin Receptor Antagonists

These drugs, particularly those targeting certain serotonin receptors (5-HT2A and 5-HT3), have shown promise in the treatment of fibromyalgia [226]. Cyclobenzaprine (5-HT2A antagonist) is a muscle relaxant that provides strong benefits in ameliorating sleep disturbances, although its effects on pain relief are only mild [227]. One additional serotonin antagonist that has garnered attention in the context of fibromyalgia is tropisetron, specifically a 5-HT3 receptor antagonist [228]. Trazodone, a drug that possesses 5-HT2A and 5-HT2C antagonist properties, has exhibited considerable advantages in the management of fibromyalgia. An investigation conducted by Morillas-Arques et al. indicates that trazodone enhanced sleep quality and reduced fibromyalgia symptoms [229]. These drugs can elicit some side effects, including nausea, dizziness, xerostomia, constipation, drowsiness, mood disorders, blurred vision, and difficulties with focus and concentration [230].

#### 6.1.6. Gabapentinoids

Pregabalin and gabapentin are two of the most important classes of drugs currently used in pain treatment; both exert their effects by binding to the α_2_δ subunit of VGCCs in the CNS. Pregabalin has received FDA approval for the treatment of fibromyalgia and is recommended in numerous guidelines [231,232]. One of the strongest meta-analyses involving RCTs suggests that pregabalin and gabapentin are effective in reducing pain, fatigue, and sleep disturbances. However, this analysis indicates that these drugs have limited effects on depressive symptoms, while their impact on anxiety is restricted [233,234]. On the other hand, mirogabalin is highly effective in reducing pain intensity and improving the quality of life in patients with fibromyalgia [235]. While these agents are crucial in pain management, their efficacy in terms of the resolution of psychological problems has yet to be proven, given the complexity and multi-dimensionality of the therapeutic landscape. Common side effects (such as dizziness, somnolence, and peripheral oedema) can significantly impact the quality of life and daily functioning of patients with fibromyalgia. However, other side effects might manifest as weight gain, cognitive impairment, and an increased risk of suicidal thoughts or behaviors [236].

#### 6.1.7. Antipsychotics

There is much evidence in support of the use of antipsychotics in treating fibromyalgia [237]. Quetiapine, a 5-HT2A and dopamine D2 antagonist, has shown considerable improvements in relation to depression, pain, and overall quality of life [238]. Olanzapine, another antipsychotic, alleviates some symptoms linked to fibromyalgia, especially when combined with depressive disorders [239]. However, the utilization of these medications is constrained by side effects, including weight gain and somnolence, which have led some patients to discontinue their treatment regimens [240]. Although these findings are based on earlier studies, further research is essential to confirm their efficacy and safety in modern clinical practice. This underscores the necessity for ongoing investigations in this domain [240].

#### 6.1.8. Dopamine Receptor Agonists

This class of drugs has received much attention in recent times, especially with the growing evidence linking dopaminergic pathways to the pathophysiology of fibromyalgia [241,242]. Agents such as pramipexole and ropinirole have shown encouraging effects in ameliorating the pain symptoms that usually characterize fibromyalgia [243,244]. At the same time, further research is highly needed in order to delineate a clear profile of the efficacy and safety of such interventions, as the variability of fibromyalgia symptoms complicates the treatment approach. The most common side effects related to the use of these drugs are nausea, vomiting, and orthostatic hypotension; these are generally more prominent during the early stages of treatment [245]. Sleep issues, like excessive daytime sleepiness and abrupt onset of sleep, are other common adverse effects. Other problems include weight loss and gastrointestinal complications, especially constipation [245]. Other severe side effects may present as impulse control disorders, such as pathological gambling, hypersexuality, compulsive shopping, and binge eating [245].

#### 6.1.9. N-methyl-D-aspartate (NMDA) Antagonists

These medications have received much attention in the management of pain. Glutamate, recognized as the principal excitatory neurotransmitter within the CNS, plays a significant role in the manifestation of chronic pain symptoms [246]. Among the three different types of glutamate receptors, the NMDA receptors are clearly involved in this pathological mechanism [246]. Consequently, this has prompted serious investigation into the potential therapeutic role of NMDA antagonists in the management of fibromyalgia [238]. Studies using ketamine have shown significant effectiveness in lowering the referred pain in patients with fibromyalgia [247]. Memantine is able to reduce neurotoxicity due to high levels of glutamate in the CNS of individuals with fibromyalgia and diminish their pain as well [248]. Side effects associated with the drug include hypertension, confusion, headache, constipation, cough, and generalized pain. Other symptoms include yawning, vomiting, and dyspnea (which is sometimes followed by severe fatigue) [249].

#### 6.1.10. Cannabinoids

Given the role of the endocannabinoid system in pain modulation, it has been hypothesized that the pathophysiology of fibromyalgia is associated with a high deficiency in endocannabinoid activity [250,251]. The two major active cannabinoids include THC and CBD [252]. THC is known for its psychoactive properties, which influence pain and emotional regulation by interacting with CB1 and CB2 receptors [251]. The potential benefits may extend beyond pain relief, with many patients also reporting enhanced sleep quality, reduced anxiety, and an improvement in the quality of life [253,254]. Of the major cannabinoid agonists under investigation, nabilone, dronabinol, and ajulemic acid have shown positive results in terms of pain relief [255,256,257,258]. The most frequent side effects associated with cannabinoid therapies include sleepiness, dry mouth, sedation, and dizziness. Other patients manifest euphoria, tachycardia, and hypotension [259]. Furthermore, there are long-term concerns such as decreased physiological functioning, tolerance development, and paranoia [259].

### 6.2. Non-Pharmacological Strategies

Non-pharmacological therapies serve as the cornerstone of managing fibromyalgia and are often considered first-line treatments due to their favorable safety profiles and potential for lasting benefits [260]. Physical exercise, mainly through programs of aerobics and strength training, has been shown to confer several advantages across a multitude of symptoms, including pain, sleep disturbances, fatigue, and depression [261]. Aerobic exercise has also been shown to significantly enhance analgesia and increase overall functionality in patients with fibromyalgia, primarily by enhancing the endogenous opioid pathways [262]. Studies advise that physical activity increases the binding affinity and activation of opioid receptors within the CNS, a phenomenon which is fundamental for effective pain modulation [262]. The physiological adaptations that occur as a result of regular exercise (such as increased β-END synthesis) contribute to elevated pain thresholds and overall analgesic effects [263].

Psychological interventions, such as cognitive behavioral therapy and mindfulness, have been identified as effective in reducing pain disturbances, addressing sleep irregularities, and mitigating depressive symptoms [264,265]. CAM techniques, including acupuncture, massage therapy, and balneotherapy, have also revealed potential benefits in managing symptoms [266,267,268]. Other promising strategies include patient health education, helping patients understand their condition and manage it appropriately [269].

Although these interventions are increasingly supported by a growing body of evidence, it has to be underlined that the quality and coverage of studies vary significantly, thus underlining the need for more rigorous research to establish the efficacy of certain treatments definitely [270]. Nevertheless, the available evidence underlines the need for including non-pharmacological interventions as cornerstones in a multidisciplinary approach to fibromyalgia management [271].

## 7. Future Research Directions

Current investigations into the complex relationship between the endogenous opioid system and fibromyalgia have yielded promising advancements; however, several limitations persist that require more attention in future studies. A limited understanding of the underlying mechanisms remains a significant challenge, as, despite substantial progress in neuroimaging and molecular biology, the precise ways in which the opioid system influences fibromyalgia are still unclear.

Future investigations must explore a more profound understanding of the molecular and cellular pathways implicated in opioid receptor activity and their contribution to the pathophysiology of fibromyalgia. Furthermore, inconsistent biomarker identification presents another obstacle, as neuroimaging technologies may offer new potential biomarkers, but the specificity and reliability of these indicators for fibromyalgia diagnosis and treatment evaluation have yet to be fully validated. This reinforces the need for future research to focus on identifying more consistent and reliable biomarkers that can be effectively utilized in clinical settings for accurate diagnosis and monitoring of treatment outcomes, although this task is inherently complex.

Other areas of interest involve the study of epigenetic influences. The examination of epigenetics within the context of opioid system functionality and its potential ramifications with respect to the heterogeneity of fibromyalgia represents an emerging area of research. However, there exists a conspicuous deficiency in thorough research concerning the interaction of genetic and environmental factors influencing opioid receptor activity and the symptomatic expression of fibromyalgia. Future investigations ought to encompass large, heterogeneous populations to scrutinize the epigenetic determinants that contribute to the diverse manifestations of fibromyalgia.

The investigation of new treatments constitutes the final major pillar of fibromyalgia research. Although preclinical and molecular studies show potential benefits, a substantial gap persists between laboratory discoveries and their clinical implementation. Future research should prioritize the integration of molecular insights with clinical trials in order to assess the efficacy of novel opioid modulators. This approach will advance the creation of personalized, evidence-based pain management strategies for fibromyalgia patients.

## 8. Conclusions

The endogenous opioid system plays an important role in the pathophysiology of fibromyalgia, a disease characterized by severe pain, fatigue, sleep disturbances, and cognitive impairment. Recent research has shown profound disturbance of the endogenous opioid system in fibromyalgia, indicating that impaired pain processing mechanisms play a critical role in the increased pain sensitivity experienced by these individuals. The endogenous opioid system, comprising opioid peptides and their receptors, plays a key role in pain perception. There is solid evidence for reduced density of opioid receptors in CNS regions involved in pain modulation in the context of fibromyalgia. This dysregulation impairs the ability to regulate pain which, in turn, exacerbates the symptoms of fibromyalgia. Although these findings provide valuable insights, more research is needed to fully understand the complex mechanisms behind this dysfunction.

The dysfunction of the opioid system in individuals afflicted with fibromyalgia may elucidate the diminished efficacy of conventional opioid therapies within this cohort. However, certain therapeutic modalities (such as low-dose naltrexone) exhibit promise in reestablishing an equilibrium within the endogenous opioid system, thereby suggesting potential in restoring the balance within the endogenous opioid system. Non-pharmacological interventions, such as cognitive behavioral therapy and physical exercise, offer an integrated approach to the different symptoms associated with fibromyalgia. While recent studies provide promising insights into the role of the endogenous opioid system in fibromyalgia, further research is needed to uncover the underlying mechanisms. This will be crucial in developing more efficient therapies that can be adapted to particular needs of fibromyalgia patients.

## Figures and Tables

**Table 1:** List of symptoms linked to fibromyalgia. Abbreviations: IBS (irritable bowel syndrome); RLS (restless legs syndrome).

Symptom Category	Specific Symptoms	References
Pain	Widespread musculoskeletal pain, hyperalgesia (heightened pain sensitivity), allodynia (pain from normally non-painful stimuli), and muscle and joint stiffness	[1,2]
Fatigue	Persistent fatigue, feeling unrefreshed after sleep, and decreased energy levels	[1,3]
Sleep disturbances	Difficulty falling asleep, frequent awakenings, and non-restorative sleep	[1,4]
Cognitive issues	Cognitive deficits (“fibro fog”), memory loss, impaired concentration, and poor vocabulary recall	[1,5]
Mood disorders	Anxiety, depression, and mood swings	[1,6]
Sensory impairments	Headaches (including migraines), paresthesias (tingling or numbness), and sensitivity to temperature, light, sound, and odors	[1,7]
Gastrointestinal disorders	Irritable bowel syndrome (IBS), abdominal pain, and bloating	[1,8]
Other symptoms	Morning stiffness, dizziness, restless legs syndrome (RLS), and painful menstrual periods	[1,9,10,11,12]

**Table 2:** A comprehensive list of the opioid receptors, including their natural ligands, physiological functions, and pathological roles.

Opioid Receptor	Endogenous Ligands	Physiological Roles	Pathological Roles	References
MOR	β-END ENKs	Analgesia, respiratory regulation, sedation, gastrointestinal motility, and euphoria	Addiction, respiratory depression, and constipation	[34,35,36,37,38,39,40,41,42,43,44,45,46]
DOR	ENKs	Analgesia, gastrointestinal motility, neuroprotection, cardioprotection, and modulation of inflammatory responses	Addiction, and role in psychiatric disorders	[35,47,48,49,50,51,52,53,54,55,56]
KOR	DYNs	Analgesia, and stress response	Dysphoria, hallucinations, and role in mood disorders	[35,57,58,59,60,61,62,63,64,65,66]

**Table 3:** List of frequently used opioid medications for treating fibromyalgia pain symptoms. Abbreviations: SNRI (serotonin and noradrenaline reuptake inhibitor); NRI (selective noradrenaline reuptake inhibitor); MOR (μ-opioid receptor).

Drug	Action Mechanisms	Principal Side Effects	References
Tramadol Tapentadol	MOR agonist with SNRI activity MOR agonist with NRI activity	Nausea, dizziness, constipation, somnolence, respiratory depression, seizures, cardiovascular effects, risk of dependence, and withdrawal symptoms	[190,191,192,193,194]
Naltrexone	Non-selective opioid antagonist	Insomnia, headaches, and nausea	[195,196,197,198]

## Abbreviations

The following abbreviations are used in this manuscript:

5-HT2A	5-HT2A receptor (serotonin)
5-HT2C	5-HT2C receptor (serotonin)
5-HT3	5-HT3 receptor (serotonin)
ACR	American College of Rheumatology
AC	Adenylyl cyclase
ACTH	Adrenocorticotropic hormone
ASIC	Acid-sensing ion channel
CAM	Complementary and alternative medicine
cAMP	Cyclic adenosine monophosphate
CB1	Cannabinoid receptor 1
CB2	Cannabinoid receptor 2
CBD	Cannabidiol
CD19	Cluster of differentiation 19
CD4	Cluster of differentiation 4
CGRP	Calcitonin gene-related peptide
CNS	Central Nervous System
D2	Dopamine receptor D2
DAG	Diacylglicerol
DOR	δ-opioid receptor
DYN	Dynorphin
ENK	Enkephalin
ERK	Extracellular signal-regulated kinase
EULAR	European Alliance of Associations for Rheumatology
FDA	Food and Drug Administration
FIQ	Fibromyalgia Impact Questionnaire
fMRI	Functional magnetic resonance imaging
GABA	γ-aminobutyric acid
GIRK	G protein-coupled inward rectifying potassium channel
GPCR	G protein-coupled receptor
HPA	Hypothalamic-pituitary-adrenal axis
IBS	Inflammatory bowel disease
IFN-γ	Interferon gamma
IL-10	Interleukin 10
IL-17	Interleukin 17
IL-6	Interleukin 6
IL-8	Interleukin 8
IP3	Inositol triphosphate
KOR	κ-opioid receptor
LDN	Low-dose naltrexone
Leu-ENK	Leu-enkephalin
M1	M1 phenotype macrophage
M2	M2 phenotype macrophage
MAPK	Mitogen-activated protein kinase
Met-ENK	Met-enkephalin
MOR	μ-opioid receptor
MYT1L	Myelin transcription factor 1 like
NMDA	*N*-methyl-D-aspartate
NGF	Nerve growth factor
NRI	Selective noradrenaline reuptake inhibitor
NRXN3	Neurexin 3
OPRD1	Opioid receptor delta 1
OPRK1	Opioid receptor kappa 1
OPRM1	Opioid receptor mu 1
P2X3	P2X purinergic receptor type 3
PBMC	Peripheral blood mononuclear cell
PC	Prohormone convertase
PDYN	Prodynorphin
PENK	Proenkephalin
PKA	Protein kinase A
PLC	Phospholipase C
PNS	Peripheral Nervous System
POMC	Proopiomelanocortin
RCT	Randomized controlled trial
RLS	Restless legs syndrome
ROS	Reactive oxygen species
SLC6A4	Solute carrier family 6 member 4
SNP	Single nucleotide polymorphism
SNRI	Serotonin and noradrenaline reuptake inhibitor
SOD	Superoxide dismutase
SSRI	Selective serotonin reuptake inhibitor
TCA	Tricyclic antidepressant
TGF-β	Transforming growth factor beta
THC	Tetrahydrocannabinol
TNF-α	Tumor necrosis factor alpha
TRPV	Transient receptor potential vanilloid
TRPV1	Transient receptor potential vanilloid 1
TRPV2	Transient receptor potential vanilloid 2
VGCC	Voltage-gated calcium channel
α-MSH	α-melanocyte-stimulating hormone
β-END	β-endorphin
β-LPH	β-lipotropin
γ-LPH	γ-lipotropin
γ-MSH	γ-melanocyte-stimulating hormone
